# Natural variability in air–sea gas transfer efficiency of CO_2_

**DOI:** 10.1038/s41598-021-92947-w

**Published:** 2021-06-30

**Authors:** Mingxi Yang, Timothy J. Smyth, Vassilis Kitidis, Ian J. Brown, Charel Wohl, Margaret J. Yelland, Thomas G. Bell

**Affiliations:** 1 Plymouth Marine Laboratory, Prospect Place, Plymouth, UK; 2 National Oceanography Centre, European Way, Southampton, UK

**Keywords:** Marine chemistry, Ocean sciences

## Abstract

The flux of CO_2_ between the atmosphere and the ocean is often estimated as the air–sea gas concentration difference multiplied by the gas transfer velocity (*K*_660_). The first order driver for *K*_660_ over the ocean is wind through its influence on near surface hydrodynamics. However, field observations have shown substantial variability in the wind speed dependencies of *K*_660_. In this study we measured *K*_660_ with the eddy covariance technique during a ~ 11,000 km long Southern Ocean transect. In parallel, we made a novel measurement of the gas transfer efficiency (GTE) based on partial equilibration of CO_2_ using a Segmented Flow Coil Equilibrator system. GTE varied by 20% during the transect, was distinct in different water masses, and related to *K*_660_. At a moderate wind speed of 7 m s^−1^, *K*_660_ associated with high GTE exceeded *K*_660_ with low GTE by 30% in the mean. The sensitivity of *K*_660_ towards GTE was stronger at lower wind speeds and weaker at higher wind speeds. Naturally-occurring organics in seawater, some of which are surface active, may be the cause of the variability in GTE and in *K*_660_. Neglecting these variations could result in biases in the computed air–sea CO_2_ fluxes.

## Introduction

The ocean has absorbed roughly a quarter to a third of anthropogenic CO_2_ emissions since the industrial revolution^1,2^. About half of the global oceanic CO_2_ uptake occurs in the Southern Ocean^3,4^—a vast, poorly observed region with areas of deepwater formation that sequesters carbon from the surface^5^. Air–sea CO_2_ flux is generally estimated as the product of the air–sea CO_2_ concentration difference and the CO_2_ gas transfer velocity (here denoted as *K*_660_). There remains substantial (at least 25%) uncertainty in the estimation of air–sea CO_2_ flux^6^, in large part due to uncertainty and variability in *K*_660_^7^. This limits our ability to accurately assess the current carbon cycle and predict future climate scenarios.

Wind provides the primary turbulent forcing for air–sea exchange by governing near surface hydrodynamics^8^. As a result, *K*_660_ is typically parameterized solely as a function of wind speed. However, mean wind speed dependencies in *K*_660_ derived from recent air–sea CO_2_ flux measurements in the Southern Ocean^9–11^ vary considerably (~ 20% at intermediate wind speeds and ~ 100% at low and high wind speeds; see supplement). Waves^12,13^, bubbles^9,14,15^, and surfactants^16–18^ (focus of this study) have been proposed as additional controlling factors for *K*_660_.

A myriad of different natural organic materials exists in the surface ocean, varying in concentration, molecular size, solubility, and surface activity. The more surface active organics, or surfactants, tend to be more concentrated near the air–sea interface (within a ca. 100 μm depth region often called the sea surface microlayer, or SML) relative to the waters below. Surfactants can be roughly divided into insoluble and soluble types, which are thought to affect gas transfer through different mechanisms. Insoluble surfactants can form film or slicks, providing additional barrier to air–sea transfer (e.g. ref.^19^). Such insoluble surfactant films are quickly dispersed by wind and waves and are thus likely to be important only during very calm conditions^20^. Soluble surfactants impact gas transfer across a wider range of wind conditions by changing the surface ocean hydrodynamics^21^. Recent surveys of surfactants in SML (a challenging measurement in situ) demonstrate that they are ubiquitously present in the global oceans with large variability in concentration (~ 60 μg L^−1^ to a few mg L^−1^ Triton-X-100 equivalent^18,22^).

Tank experiments show that naturally occurring surfactants can suppress the rate of gas transfer by 10–50% (e.g. oxygen by ref.^23,24^; methane by ref.^17,25^). A direct relationship was reported between gas transfer suppression in a tank and the surfactant concentration in SML waters^17,25^. Organic materials excreted from marine phytoplankton, including carbohydrates associated with proteins and possibly lipids, appear to be the principal classes of molecules responsible for gas transfer suppression^16^. The main drawback of these studies is that the nature of turbulence in the upper ocean cannot easily be compared with the turbulence in the tanks (e.g. induced by a baffle in ref.^25^, and by a shaker table in ref.^16,23^).

Field evidence of gas transfer suppression by natural surfactants is very limited. Frew et al.^24^ inferred *K*_660_ from a thermal imaging technique in coastal waters at low wind speeds. They found that in the presence of natural surfactant films (identified by surface enrichment in the absorption of coloured dissolved organic matter, CDOM), the inferred gas transfer velocity was lower by up to an order of magnitude relative to the no-film case. Mustaffa et al.^18^ coupled measurements of surfactant concentration with estimates of CO_2_ transfer using a floating chamber at wind speeds less than 7 m s^−1^ in different parts of the Atlantic and Pacific oceans as well as in nearshore waters. Their measurements suggest a relative gas transfer suppression of 23% (sufficient surfactant present in SML, but no film) to 62% (surfactant film). It is unclear from these observations what the effect of surfactants might be on near surface hydrodynamics and hence on *K*_660_ at moderate to high wind speeds.

Here we directly measured the air–sea CO_2_ flux using the eddy covariance method and the seawater fugacity of CO_2_ during a recent shipboard transect in the Southern Ocean. This enabled us to derive *K*_660_ at wind speeds up to 18 m s^−1^. In parallel, we present a novel measurement of the gas transfer efficiency (GTE) based on partial equilibration of CO_2_ using a purpose-built, dual Segmented Flow Coil Equilibrator (SFCE) system. This system provides a quantification of the effect of varying seawater composition on gas transfer that is not directly driven by wind. We combine these observations to better understand the hydrodynamical processes that drive air–sea CO_2_ transfer.

## Methods

### Eddy covariance measurements of air–sea CO_2_ flux

Eddy covariance (EC) is the most direct method for observing the air–sea CO_2_ flux and its application to shipboard measurements has been improved significantly in recent years^26–28^. Briefly, the CO_2_ flux is determined by correlating high frequency (here 10 Hz) fluctuations in the dry mixing ratio of atmospheric CO_2_ (xCO_2_) with those in the vertical wind velocity (*w*) and averaging over time (here 20 min): $\overline{{xCO_{2} ^{\prime } w^{\prime}}}$. Measurements of winds on a ship are affected by the ship’s motion and by flow distortion due to the ship’s superstructure^29^. A sonic anemometer (Metek uSonic-3 Scientific) measuring 3D wind velocities as well as a motion sensor measuring 3D linear accelerations and rotational rates (Systron Donner Motionpak II) were installed on the foremast of the RRS James Clark Ross (JCR), 21.5 m above water. The motion data were used to correct the wind measurements for the ship motion following established methods^12,26,30^, while three dimensional computational fluid dynamic modeling results for the JCR^31^ were used to correct the measured mean wind speed for flow distortion. The COARE 3.5 model^32^ was used to convert the observed true wind speed to 10-m neutral wind speed (*U*_10*n*_). The 10-m neutral drag coefficient derived from eddy covariance (u_*_^2^/*U*_10*n*_^2^) closely agrees with the COARE 3.5 model, suggesting that both the motion and the flow distortion corrections are reasonable (Supplementary Fig. S1).

The CO_2_ air intake was mounted 73 cm below the center volume of the Metek sonic anemometer, and the sample air was pulled rapidly through a 30 m length of 9.5 mm inner diameter (ID) Teflon tube by a dry vacuum pump (Gast 1023 series) at a flow rate of ~ 40 L a minute (LPM). A Picarro Cavity Ringdown Spectrometer (G2311-f) sub-sampled from the main inlet tube for the dry CO_2_ mixing ratio at ~ 5 LPM. Before entering the Picarro, sample air passed through a short section of 3.2 mm ID Teflon tube, a particle filter (2 μm), and a counter-flow dryer (Nafion PD-200T-24M). The dryer minimized the interference in CO_2_ measurement due to water vapour (H_2_O) by removing ~ 80% of the H_2_O in the mean and almost all of the variability^33^. The H_2_O signal was measured concurrently by the Picarro instrument and was used to numerically remove the residual H_2_O signal within the Picarro software^34^, yielding the atmospheric CO_2_ dry mixing ratio (xCO_2_, in ppm) needed for the flux calculation.

Measuring CO_2_ downstream of a long inlet tube and a dryer resulted in a delay in the CO_2_ signal relative to the wind measurement and a small amount (ca. 10%) of high frequency attenuation in the flux signal. A puff of nitrogen gas was injected into the inlet tip once every 6 h. The decay in the gas signal (due to dilution by the nitrogen) was used to estimate both the delay time (3.4 ± 0.2 s) and the response time (0.36 s). The delay in the CO_2_ signal is accounted for in the flux calculation, while the high frequency flux attenuation is corrected with a filter function approach using the measured response time (Landwehr et al. 2018). The Picarro instrument is sensitive to motion acceleration, yielding substantially higher variance in xCO_2_ when the ship is in rough seas. The heave of the ship also means that the sample inlet moves vertically along a gradient in xCO_2_^26^. These motion effects are corrected for by decorrelating xCO_2_ measurements with the ship’s acceleration, velocity, and displacement^12^. The flux in mixing ratio units (e.g. ppm m s^−1^) is converted to molar fluxes (e.g. mmole m^−2^ day^−1^) using the dry density of air computed from air temperature, pressure, and humidity measurements.

The wind sector of ± 130 degrees (0 degree for directly over the bow) is considered for fluxes, excluding periods of contamination from ship’s exhaust when the winds were from the aft. Additional quality control criteria for CO_2_ flux are similar to ref.^12^, and further include stationarity measures of wind^35,36^. To reduce random noise, quality controlled 20-min fluxes are averaged to hourly intervals or averaged in wind speed bins (width of 2 m s^−1^). The uncertainty in EC CO_2_ flux is estimated empirically and propagated to *K*_660_ (~ 20% for an hourly average). See Dong et al.^37^ for further details on flux processing and uncertainty analysis.

### Seawater fCO_2_ and calculation of CO_2_ gas transfer velocity (*K*_660_)

Measurements of high-resolution underway fCO_2_ and gas transfer efficiency were made using two Segmented Flow Coil Equilibrator (SFCE) system deployed in parallel on the ANDREX II cruise. fCO_2_ was additionally measured with a standard vented-showerhead equilibrator system coupled to an infrared gas analyzer^38^. The SFCE, modified from earlier designs^12,39^, has been described in detail by Wohl et al.^40^. Briefly, ship’s underway seawater (from ~ 6 m depth) was piped into a ~ 200 mL glass bottle via a ~ 1 m long 6.4 mm ID Teflon tube and allowed to overflow into the sink. For each SFCE, water was extracted from the bottom of this glass bottle by a peristaltic pump via a ~ 0.5 m long 4.0 mm ID Teflon tube and a ~ 15 cm long 4.4 mm ID Pumpsil soft, platinum-cured silicon tube. CO_2_-free synthetic air was added continuously to the sample seawater at a Teflon ‘tee’ piece, naturally forming distinct, cm-long water and air segments. The air and water segments traveled in the same direction through a 4.0 mm ID Teflon tube, where gas transfer occurs. Water and air segments were then separated at an air–water separating ‘tee’. Sample air left from the top of the separator and flowed towards the CO_2_ analyzer (Licor7000), while the sampled water was drained away from the bottom of the separator. The two SFCEs operated simultaneously and alternate CO_2_ measurements were made with the same Licor7000 every five minutes (switching controlled by a solenoid valve). The water flow rate was controlled for each SFCE at 100 mL min^−1^ by the peristaltic pump (flow monitored several times a day). The total synthetic airflow rate was set at 50 mL min^−1^ by a mass flow controller, which was split evenly between the two SFCEs. Sample air was dried with a Nafion dryer and filtered with a Swagelok particle filter to reduce the influence of humidity and particulates on the CO_2_ measurement. Compared to membrane-based equilibrators, advantages of the SFCE include rapid gas transfer (due to surface renewal within individual segments^39^) and the absence of membranes that could become clogged due to bio-fouling. As a precautionary measure, the SFCEs were washed with 10% hydrochloric acid every few days to prevent any internal biological growth.

The two SFCE systems were identical except for the length of the gas transfer coil (20 m vs. 40 cm). The long coil was kept close to ambient sea surface temperature by immersion in a rapidly overflowing bucket of underway seawater. Continuous monitoring indicated that the bucket water temperature was consistently 1 °C higher than ambient water temperature, and this temperature difference was accounted for in the fCO_2_ calculation below for thermally induced change in carbonate chemistry^41^. Measurement from the long coil (CO_2_long_) is related to the dry CO_2_ mixing ratio at equilibrium with ambient seawater (xCO_2w_) by a purge factor (*PF*): xCO_2w_ = CO_2_long_ *PF*. Purging occurs because gas exchange with CO_2_-free air reduces CO_2_ in the aqueous phase, such that CO_2_long_ is lower than xCO_2w_. We define *PF* as the aqueous concentration before equilibration divided by the aqueous concentration after equilibration. At the low temperatures encountered during this transect (0.7 ± 0.5 °C), *PF* for underway seawater is well predicted by mass conservation^40^:

1 $$PF = {\text{ 1 }} + {\text{ 1}}/\left( {H~Q_{w} /Q_{a} } \right).$$

Here *Q*_*w*_ and *Q*_*a*_ indicate the flow rates of water and air, respectively, and *H* is the dimensionless solubility of CO_2_ (water to air). For 0.7 °C seawater (cruise mean), *PF* was 1.176 for CO_2_ at a water flow rate of 100 mL min^−1^ and airflow rate of 25 mL min^−1^.

Following ref.^41^, xCO_2w_ derived from the long coil is converted to seawater fCO_2_. Seawater fCO_2_ derived from the long coil SFCE and from the widely used showerhead equilibrator demonstrate exceptionally good agreement during the ANDREXII cruise (Supplementary Fig. S2). This suggests near full equilibration of CO_2_ within the long coil as well as high stability of the SFCE system. For the calculation of *K*_660_, we use seawater fCO_2_ from the showerhead equilibrator where available and use fCO_2_ from the SFCE to fill any remaining gaps (see Fig. S2). Atmospheric fCO_2_ (mean ± standard deviation of 390.6 ± 6.6 μatm) was subtracted from seawater fCO_2_ to yield ΔfCO_2_. Maps of the transect colour-coded by CO_2_ flux and ΔfCO_2_ are shown in Supplementary Fig. S3.

The CO_2_ transfer velocity was computed as follows to facilitate comparison with previous measurements: *K*_*CO2*_ = flux/*S*/ΔfCO_2_. Here *S* is the dimensional CO_2_ solubility as a function of temperature and salinity^42^. *K*_*CO2*_ is scaled to a Schmidt number of 660 assuming an exponent of − 0.5: *K*_660_ = *K*_*CO2*_ (660/*Sc*)^−1/2^, where *Sc* is the ambient Schmidt number of CO_2_^42^. To reduce random noise as well as any bias in *K*_660_ generated by dividing by very small ΔfCO_2_, only *K*_660_ data with |ΔfCO_2_|> 30 μatm are retained.

### Derivation of the CO_2_ gas transfer efficiency (GTE)

The short coil SFCE system had a 40 cm long equilibrator tube. This length was purposely chosen such that CO_2_ did not come to full equilibration in the short coil. GTE is calculated simply as the ratio between the measured CO_2_ from the short coil (what has been transferred) and xCO_2w_ (the initial potential for transfer). Triplicate measurements of the same water demonstrate that the precision in the GTE measurement is within 0.01. Our measurement setup was kept unchanged (including the geometry of the SFCEs and air/water flow rates) and the ambient seawater temperature was nearly constant (0.7 ± 0.5 °C) during this transect. Any variations in GTE were thus most likely driven by natural variability in seawater composition. Conceptually our measurement shares some similarities to those by ref.^17,25^. Unlike those earlier observations, we do not reference GTE against pure water here, which avoids the difficulties associated with generating surfactant-free water.

Our underway measurement of GTE uses subsurface seawater (~ 6 m depth) rather than SML water. SML water would arguably be more directly relevant to air–sea transfer, although subsurface and SML water compositions have been shown to be closely coupled for most organic materials^43,44^ and for surfactant concentration^18,22^. The continuous sampling of subsurface water is much easier logistically and enables a substantially larger dataset to be generated while avoiding the many possible artifacts from SML sampling^45,46^. Discrete samples of deep seawater (typically below 1000 m) from the CTD rosette, generally thought to be devoid of labile organic compounds, were measured opportunistically (N = 8) for GTE. At approximately the same temperatures as the surface waters, these additional deepwater samples provide a reference for comparison for the underway GTE observations.

## Results

### Variability in gas transfer efficiency (GTE) and CO_2_ gas transfer velocity (***K***_660_)

**Figure 1:**
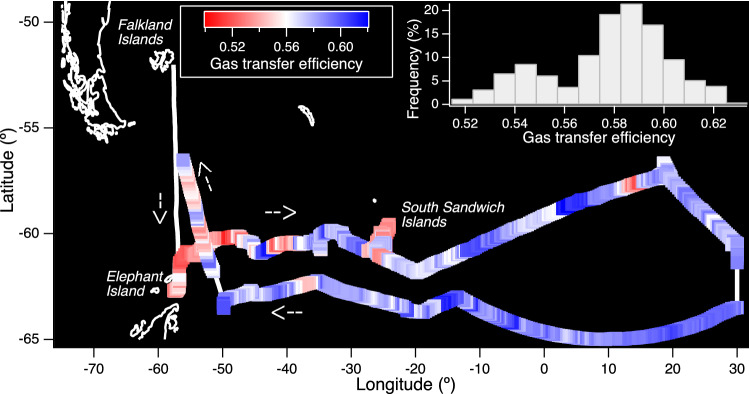
Cruise track colour-coded by the underway gas transfer efficiency, with the thick white line indicating no GTE measurement. The transect started and finished at the Falkland Islands. The return (westward) leg is displaced 2 degrees latitude south of the actual transect for clarity. GTE demonstrated substantial variability (see Supplement for further details) and a bimodal frequency distribution (inset).

Figure 1 shows the track of the ANDREXII cruise (Feb–Apr 2019), colour-coded by the underway GTE. The mean (standard deviation) GTE was 0.573 (0.024), with a range of 0.507 to 0.623 (~ 20%). GTE displayed considerable variability on short temporal and small spatial scales that often coincided with water mass changes (see supplement). The frequency distribution of GTE was bimodal—the more frequent mode was associated with higher GTE, while the less frequent mode was associated with lower GTE. Low GTE values were observed on multiple occasions and were not limited to a single region (Fig. 1).

**Figure 2:**
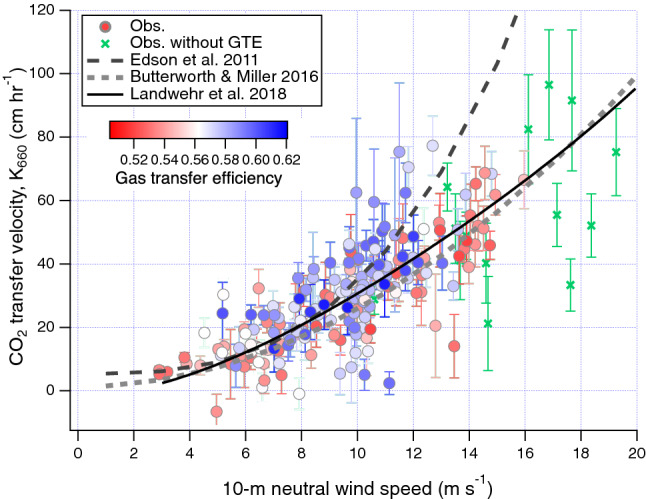
Air–sea CO_2_ transfer velocity (hourly; n = 199) vs. 10-m neutral wind speed, scaled to a Schmidt number of 660 with an exponent of − 0.5 and colour-coded by GTE. *K*_660_ observations without concurrent GTE measurements are denoted with crosses. *K*_660_ tends to be reduced when GTE was low, and vice versa. Also shown are the wind speed dependences from three direct measurements of CO_2_ *K*_660_, from ref.^9–11^. The error bars on *K*_660_ are propagated from the empirically estimated EC flux uncertainty (see ref.^37^).

Observations of the CO_2_ gas transfer velocity (*K*_660_) are shown in Fig. 2 as a function of wind speed, along with mean relationships identified during three recent CO_2_ air–sea gas transfer studies in the Southern Ocean. At moderate to high wind speeds, our observations span all of the previous wind speed relationships, but are in closer agreement in the mean (within ~ 20%) with ref.^9–11^. Wind speed explains 58% of the variance in *K*_660_ from this transect (*K*_660_fit_ =  − 0.35 + 1.10*U*_10*n*_^1^^46^; see Supplementary Fig. S4). We note that even for observations with very high signal:noise ratios (e.g. ref.^11,47^), the R^2^ value between *K*_660_ and wind speed is at most around 0.8, implying that at least 20% of the variance in *K*_660_ may be due to factors other than wind speed. While averaging in wind speed bins helps to reduce the random uncertainty in *K*_660_, doing so likely masks the variability in gas transfer caused by these other processes, which we explore next.

### Relationship between ***K***_660_ and GTE

**Figure 3:**
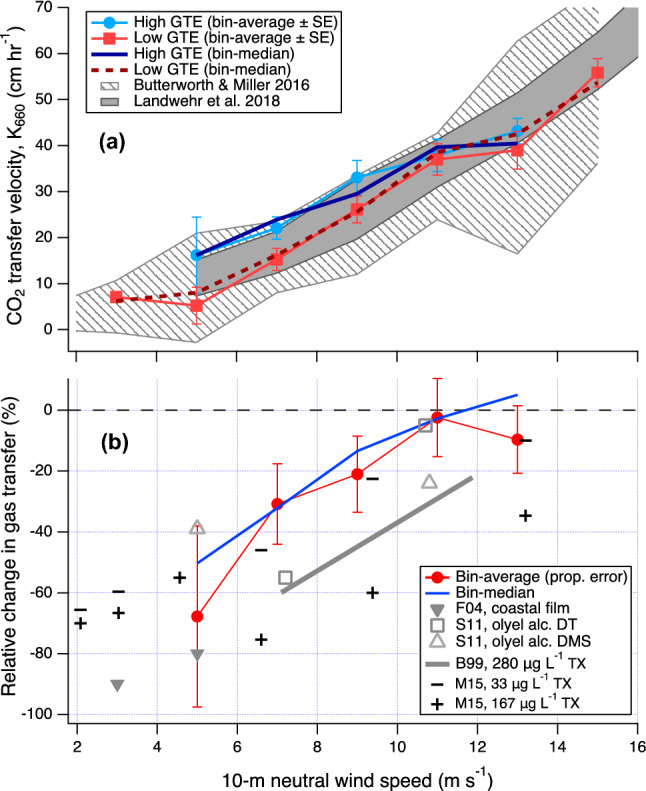
(**a**) *K*_660_ vs. 10-m neutral wind speed, separated according to high and low GTE (mean and medium in wind speed bins). *K*_660_ with high GTE clearly lies above *K*_660_ with low GTE. To illustrate the variability in previous observations, results from ref.^10,11^ are shown as bin average ± 1 standard deviation (computed from hourly data). (**b**) Relative change in *K*_660_ explainable by GTE, which was greatest at low wind speeds and diminished towards high wind speeds during this transect. Also shown are previous estimates of gas transfer suppression due to surfactants based on heat transfer measurements in coastal waters (F04; ref.^24^), based on two types of gas transfer measurements in patches of an artificial insoluble surfactant (S11; ref.^48^), and based on measurements in laboratory wind-wave tanks with a soluble surfactant (B99 and M15; ref.^49,50^). For these earlier measurements, relative suppression was computed as ratio in transfer velocity between surfactant-covered and cleaned waters, and *U*_10*n*_ was converted from the reported friction velocity where necessary using the COARE 3.5 model.

*K*_660_ in Fig. 2 is colour-coded by GTE to illustrate the effect of varying hydrodynamics. It appears that at a given wind speed, *K*_660_ values associated with high GTE tend to cluster above *K*_660_ values associated with low GTE—an effect most obvious at moderate wind speeds. To examine this further, we separate *K*_660_ measurements into groups of “high GTE” (above 0.582) and “low GTE” (below 0.540). These thresholds correspond to the peaks of the two modes in Fig. 1, and yield about a quarter of all *K*_660_ measurements in both the high GTE and low GTE groups. The bin-average and bin median of *K*_660_ associated with high GTE and low GTE are shown in Fig. 3a, with both groups largely residing within the variability of recent *K*_660_ observations by ref.^10,11^.

At wind speeds below 11 m s^−1^, the average *K*_660_ in the high GTE group is clearly greater than the average *K*_660_ in the low GTE group. We can estimate the relative effect of varying seawater composition and hydrodynamics on gas transfer from the ratio of low GTE *K*_660_ to high GTE *K*_660_: 100% (*K*_660lowGTE_/*K*_660highGTE_ − 1). As shown in Fig. 3b. the greatest relative change in *K*_660_ explainable by GTE was observed at low wind speeds (at least 50% at wind speeds below 5 m s^−1^) and this change was reduced to ~ 30% at the global mean wind speed (~ 7 m s^−1^). At wind speeds above ~ 11 m s^−1^, *K*_660_ was no longer obviously sensitive to GTE.

How does our measure of relative change in *K*_660_ explainable by GTE compare against earlier estimates of gas transfer suppression due to the known presence of surfactants? Measurements of natural waters in a tank on a North–South transect through the Atlantic Ocean (45° N to 40° S) suggest up to 32% relative gas transfer suppression due to surfactants^17^. Chamber measurements in other marine environments show a 23% relative gas transfer suppression when the SML surfactant concentration exceeds a threshold of 200 μg L^−1^ Triton-X-100 equivalent, and a further 62% suppression in the presence of surfactant films (over 1 mg L^−1^ Triton-X-100 equivalent)^18^. Field observations by Frew et al.^24^ were made in the presence of coastal surfactant films at very low wind speeds, suggesting significant contributions from insoluble surfactants. Brockmann et al. (ref.^51^) and Salter et al. (ref.^48^) released an artificial insoluble surfactant (oleyl alcohol) over km^2^-sized patches in the North Sea and in the northeast Atlantic Ocean, respectively. The surfactant additions resulted in a ~ 30% reduction in CO_2_ transfer inside of a floating chamber^51^ and up to 55% and 39% reductions in the transfer of ^3^He/SF_6_ (dual tracer) and dimethyl sulfide (DMS)^48^. In wind-wave tanks, Bock et al. (ref.^49^) and Mesarchaki et al. (ref.^50^) added a soluble surfactant (Triton-X-100) at bulk concentrations within the range of open ocean surfactant observations^18,22^; higher concentrations of Triton-X-100 generally resulted in greater gas transfer suppression.

These field and laboratory estimates of gas transfer suppression, regardless of methods employed or surfactants studied, show broadly similar magnitudes as well as similar wind speed dependencies compared to our measurements (Fig. 3b). This implies that the variation in GTE from this transect could also be due to changing natural surfactants. The relative gas transfer suppression decreases with increasing wind speed probably because at least two factors are important in determining the impact of surfactants on near surface hydrodynamics and on gas transfer: molecular composition (e.g. surfactant speciation and concentration) of the near surface water as well as physical turbulence. The GTE measurement captures the range of composition in the subsurface water at a fixed turbulence level within the SFCE system (i.e. independent of wind speed) but this is an inexact representation of the surfactants at the air–sea interface. The persistence of surfactants in the SML depends in part on the mechanism(s) available to transfer them towards/away from the interface. Gas transfer suppression by surfactants is greatest at low wind speeds, when surfactants can accumulate more easily. Wind-driven turbulence and breaking waves seem to be responsible for both the dispersion of the SML and its replenishment via mixing and rising bubbles^14,52^. In high winds, the degree of suppression is reduced, probably because surfactants at the air–sea interface are dispersed faster than they are replenished.

Theory and laboratory studies (e.g. ref.^53,54^) suggest that in very low winds or at high surfactant concentrations, the Schmidt number scaling in *K*_660_ follows an exponent that is closer to − 2/3 (suitable for a “smooth” surface) than − 1/2 (suitable for a “rough”, free surface and almost universally applied for the open ocean). For this transect, the difference between (660/*Sc*)^−1/2^ scaling and (660/*Sc*)^−2/3^ scaling amounts to about 20%, which is less than our estimate of relative change in *K*_660_ explainable by GTE at low to moderate wind speeds. This suggests that surfactants may not only change the sea surface characteristics between “smooth” and “rough”, but also alter the amount of turbulence near the sea surface.

### Implications for air–sea gas transfer and estimates of global CO_2_ flux

The Southern Ocean observations here show that at low-to-moderate wind speeds, the wind speed dependencies in *K*_660_ can vary by 30% or more, depending on seawater composition. Given the large variability in surfactants both in space^18,22^ and in time^25^, *K*_660_ derived from a single area in a single season is unlikely to be representative of the global ocean. Mustaffa et al. (ref.^18^) speculated on the global implications of surfactants on air–sea gas exchange, focusing primarily on two aspects: (1) generally lower surfactant concentration and less gas transfer suppression in the open ocean compared to nearshore waters, and (2) the occurrence of surfactant films. Considering the combined effect of wind speed and surfactants on near surface hydrodynamics, how significant are the findings of this study for global/regional air–sea CO_2_ flux estimates?

Our data do not necessarily imply that the current global air–sea CO_2_ flux should be adjusted downwards in magnitude, but highlight the uncertainty in our understanding. Widely-used parameterizations of *K*_660_ based on artificially released tracers (e.g. ref.^47,55^) or the imbalance of radiocarbon (e.g. ref.^42,56^) incorporated data from many locations/seasons and, to some extent, encompassed a range of biogeochemical and surfactant conditions. Even so, applying these parameterizations to estimate the global or regional CO_2_ fluxes may still lead to biases due to surfactants. This is because the spatial variation in surfactant concentration is very large^18,22^ and the biogeochemical controls of surfactants are not well understood. Furthermore, the air–sea CO_2_ concentration difference and winds are spatially inhomogeneous and seasonally asynchronous. For example, the tropical oceans tend to have lower wind speeds and are regions of net CO_2_ outgassing to the atmosphere, whereas the mid/high latitudes tend to have higher wind speeds and are net sinks of atmospheric CO_2_. Stronger relative gas transfer suppression in the tropics due to surfactants (whether linked to lower wind speed—see Fig. 3b, or to higher temperatures—see ref.^17^) could impede the outgassing of oceanic CO_2_ and potentially lead to a greater net global CO_2_ uptake than the current estimate (as postulated also by ref.^57^). We note here that the enhanced suppression of gas transfer in warmer waters suggested by Pereira et al. (ref.^17^) was not observed by Mustaffa et al. (ref.^18^). The very small temperature range in these Southern Ocean observations (0.7 ± 0.5 °C) precludes any investigation of temperature dependence in the GTE-*K*_660_ relationship.

The role of biogeochemistry in determining the natural surfactant abundance remains poorly understood. Our measurements of GTE in presumably organic-depleted deep seawater show a mean (standard deviation) value of 0.632 (0.029). GTE in the near surface water is on average 10% lower than in deep water, which is consistent with biological or light driven surfactants sources. However, we did not find any strong relationships between underway GTE and bulk surface biological and physical parameters (see Supplementary Figs. S5–S7), similar to findings by Goldman et al. (ref.^23^). Wurl et al. (ref.^46^) found that surfactant concentration tends to be higher in more biologically productive waters. Calleja et al. (ref.^58^) observed reduced CO_2_ transfer from a floating chamber over the open ocean in association with higher concentrations of total surface organic matter concentration. Nightingale et al. (ref.^59^) presented contrasting evidence, observing no clear reduction in *K*_660_ during the development of a large algal bloom in the equatorial Pacific. The gas transfer suppression observed by Pereira et al. (ref.^17^) was more pronounced in oligotrophic, rather than biologically productive, waters. No relationship using all cruise data was observed between surfactant concentration and either chlorophyll a concentration (at the time of sampling/2 weeks prior to sampling) or primary production in the Atlantic, possibly because of the influences of bacterial activity^22^ or photochemical processing^52^. Our work shows that in situ GTE measurements, in combination with direct fluxes by eddy covariance flux observations, provide additional insight into the variance in the gas transfer velocity vs. wind speed dependence. Studies that combine these measurements along with observations of surfactant concentration and other supporting biogeochemical parameters are needed to elucidate the effect of natural organics on gas transfer.

## Supplementary Information

Supplementary Information.
