# Supplementary material for: Natural variability in air–sea gas transfer efficiency of CO2
Source: Sci Rep. 2021 Jun 30;11:13584. doi: 10.1038/s41598-021-92947-w (PMC8245487; doi:10.1038/s41598-021-92947-w)
Supplement: Supplementary file 1 — Supplementary Information. [file 41598_2021_92947_MOESM1_ESM.pdf]

Supplementary information for:

Natural variability in air-sea gas transfer efficiency of CO_2

Mingxi Yang*[1], Timothy J. Smyth[1], Vassilis Kitidis[1], Ian J. Brown[1], Charel Wohl[1], Margaret J. Yelland[2], Thomas G. Bell[1]

1. Plymouth Marine Laboratory, Prospect Place, Plymouth, United Kingdom

2. National Oceanography Centre, European Way, Southampton, United Kingdom

*Correspondence to: miya@pml.ac.uk

Supplementary information

The eddy covariance (EC) friction velocity (u_*) was computed including both streamwise and cross stream components. The 10-m neutral drag coefficient derived from EC (u_*^2 / U_{10n}^2) closely agrees with the COARE 3.5 model[1], suggesting that both the motion correction and flow distortion correction are reasonable (Supplementary Figure S1). U_{10n} estimated using the eddy covariance friction velocity and assuming a semi-log wind profile (e.g. Eq 5-6 from ref. [2]) agrees very well with U_{10n} estimated using the COARE 3.5 model (slope = 1.0009; intercept = -0.06 m s^{-1}; r^2 = 0.996).

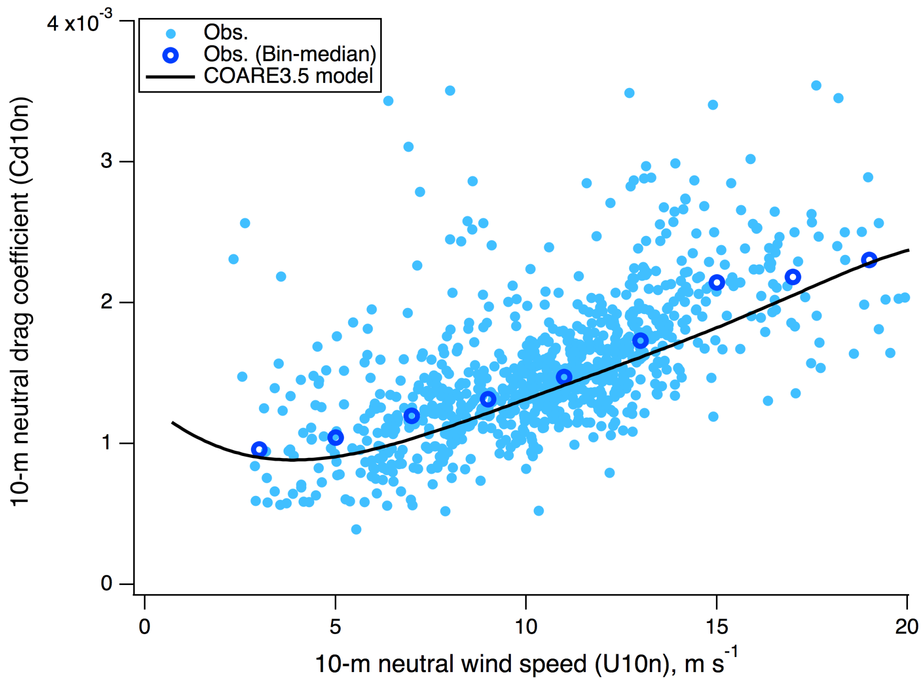

Figure S1. Measured 10-m neutral drag coefficient shows good agreement with the COARE3.5 model, validating the motion correction as well as flow distortion correction.

Seawater fCO_2 derived from the SFCE and from the widely used showerhead equilibrator demonstrate exceptionally good agreement during the ANDREXII cruise (Supplementary Figure S2). This confirms the full equilibration of CO_2 within the long coil as well as the stability of the SFCE system. For further details about this cruise, please see ref. [3].

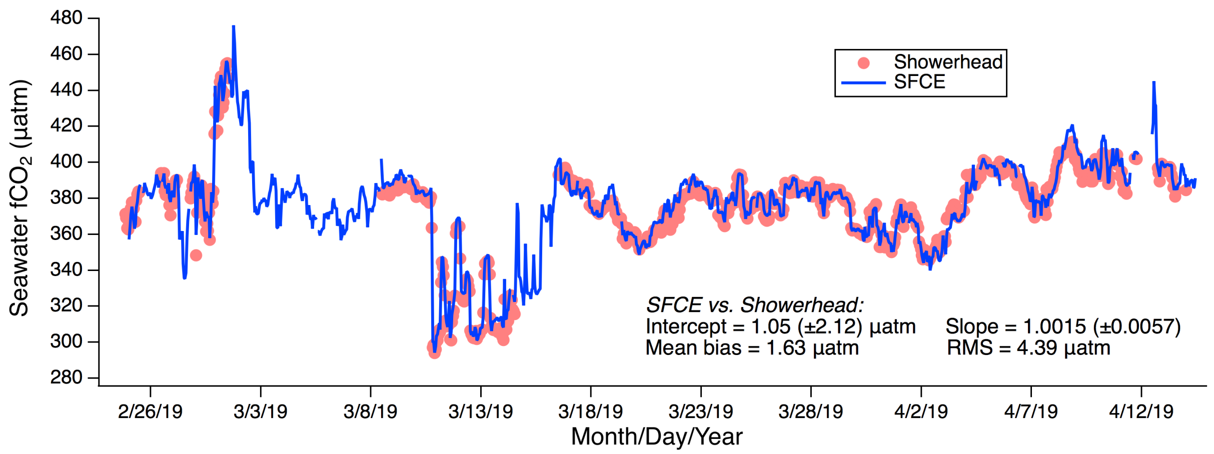

Figure S2. fCO_2 from SFCE (long coil) and from the showerhead agree exceptionally well.

CO_2 flux averaged -5.5 mmol m^{-2} d^{-1}, varying from about 21.8 mmol m^{-2} d^{-1} (net outgassing) near the Elephant Island and -43.8 mmol m^{-2} d^{-1} (net uptake) near the South Sandwich Islands (Figure S3). ΔfCO_2 showed a similar spatial pattern, ranging between +76.2 and -91.2 µatm at those locations, respectively. K_{660} associated with low GTE (Figure 3) were observed near both the Elephant Island and the South Sandwich Islands. K_{660} associated with high GTE were observed mostly further east.

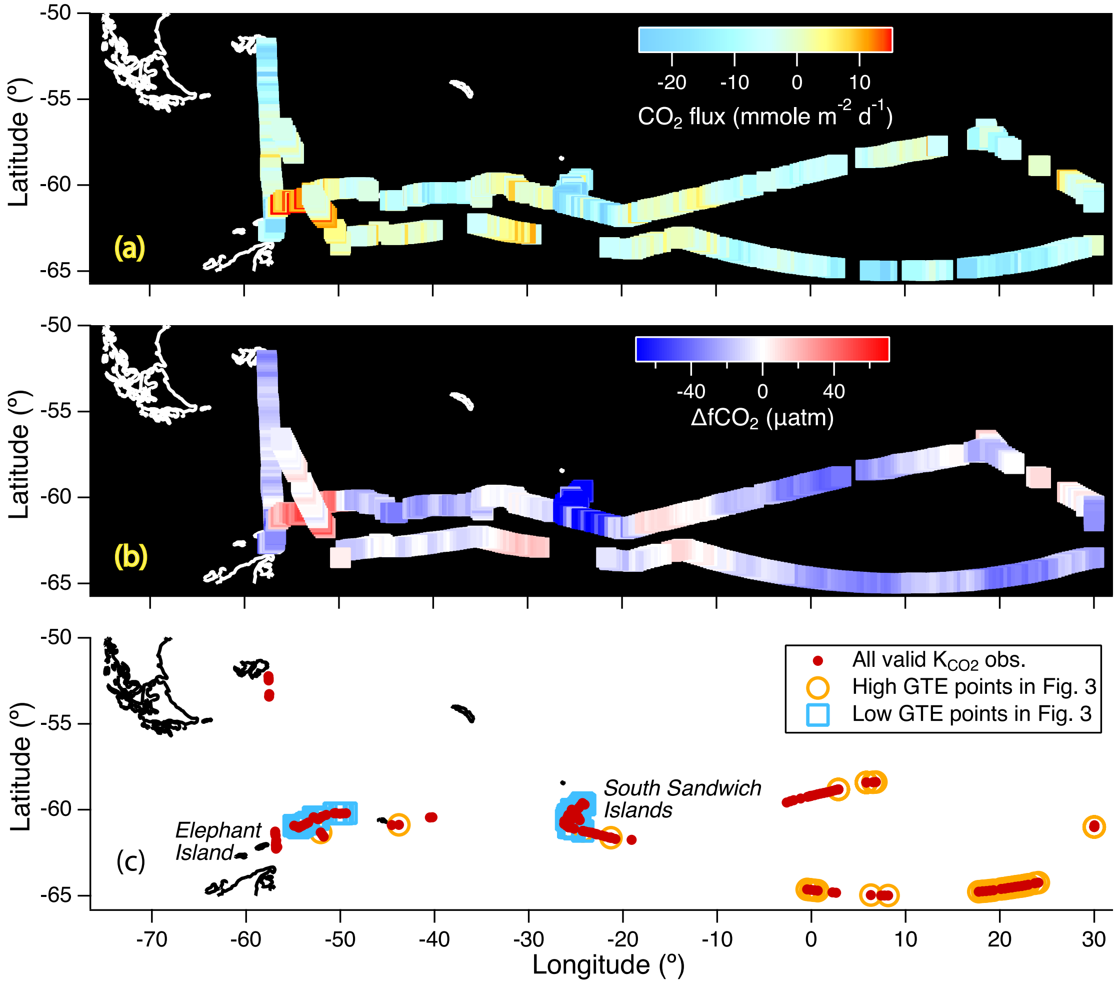

Figure S3. Cruise track color-coded by a) directly measured air-sea CO_2 fluxes, b) air-sea difference in CO_2 fugacity, ΔfCO_2, and c) locations of the quality controlled K_{CO2} measurements. Note that the color-scales are limited for visualization, as the full ranges in flux and ΔfCO_2 were -44 to +22 mmol m^{-2} d^{-1} and -94 to +76 µatm, respectively. The transect started and finished at the Falkland Islands. The return (westward) leg is plotted 2 degrees latitude south of the actual transect for clarity. CO_2 flux was mostly negative (ocean uptake), with strong absorption near the South Sandwich Islands and large outgassing near the Elephant Island. Low GTE values with concurrent valid K_{CO2} measurements were found near both Elephant Island and the South Sandwich Islands.

The bin-average of all valid K_{660} data from this transect is shown in Supplementary Figure S4a, along with other recent measurements in the Southern Ocean. Our K_{660} observations are reasonably well fitted by a power function of 10-m neutral wind speed, U_{10n} ($K_{660\_fit} = -0.35 + 1.10U_{10n}^{1.46}$; $R^2 = 0.58$). Excluding the low GTE values in Figure 3, the fit to our data is slightly higher: $-0.11 + 1.12U_{10n}^{1.46}$.

The comparability among these datasets at different wind speeds is more clearly illustrated by computing the dimensionless Dalton number (K_{660} / U_{10n}), which is shown in Figure S4b. While bin-averaging helps to reduce the random uncertainty in K_{660}, doing so likely masks the variability in gas transfer due to other factors such as surfactants, which the GTE measurement helps to elucidate.

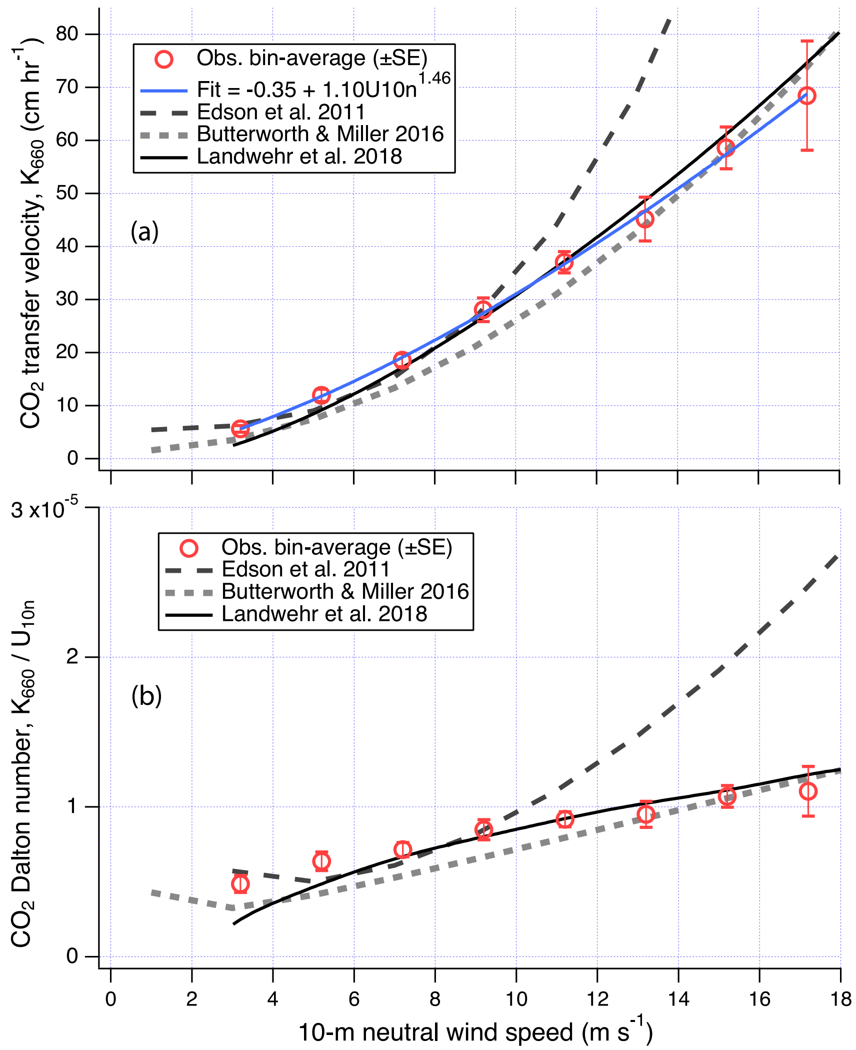

Figure S4 (a). Bin-average of the observed CO_2 transfer velocity (cm hr^{-1}), normalised to Sc=660 using an exponent of -0.5, along with a power fit as a function of 10-m neutral wind speed, U_{10n} in m s^{-1} (K_{660_fit} = -0.35 + 1.10$U_{10n}^{1.46}$ U_{10n}^{2}). Wind speed dependencies from three recent direct measurements of CO_2 K_{660} are also shown: Butterworth & Miller (2016) (1.3 + 0.245 U_{10n}^{2}; ref. [4]); Edson et al. 2011 (5.4 + 0.029 U_{10n}^{3}; ref. [5]); and Landwehr et al. 2018 (-7.3 + 104.8 u_*, here converted to U_{10n} using the COARE3.5 model; ref. [6]). b) Same data as in (a) but converted to the dimensionless Dalton number (K_{660} / U_{10n}) and again plotted against U_{10n}.

GTE demonstrated noticeable spatial and temporal variability. A short time series of SFCE measurements is shown in Supplementary Figure S5a. A brief decrease in seawater xCO_{2w} occurred between about 0100 and 0300 on 28 February. Interestingly, GTE also decreased during this period. As shown in Figure S5b, temperature and chlorophyll a concentration changed markedly during this period, indicating that the ship was briefly in a distinct watermass.

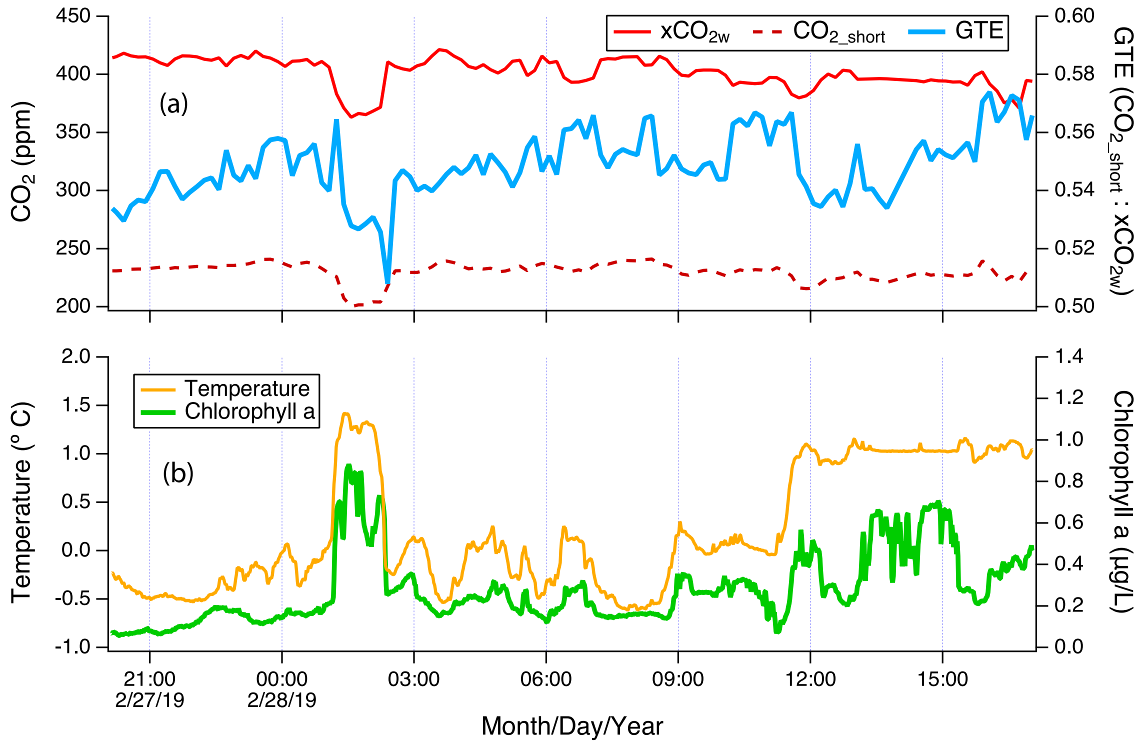

Figure S5 a) short time series of xCO_2 from the long and short coils, as well as gas transfer efficiency (GTE, computed as the ratio between the short coil and long coil); b) underway Chlorophyll a and water temperature during the same period.

A temperature-salinity diagram (Supplementary Figure 6) shows that low GTE were observed near (but not limited to) both Elephant Island and the South Sandwich Islands.

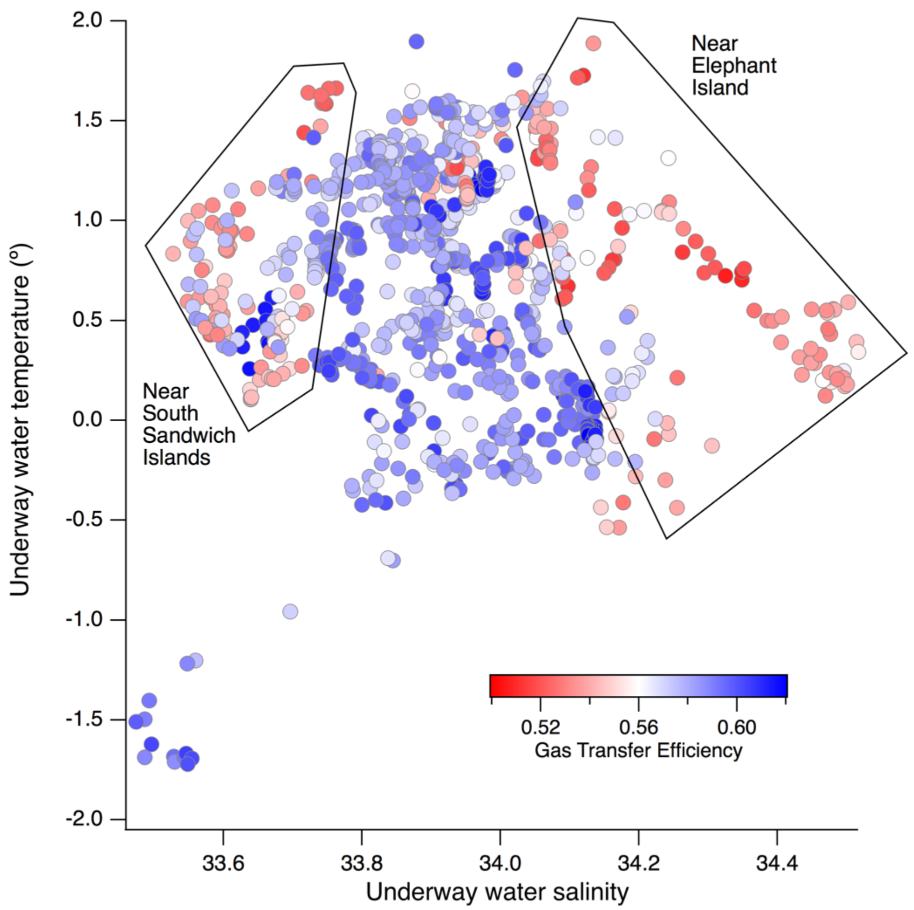

Figure S6. Temperature-salinity diagram color coded by GTE.

GTE correlated very weakly against bulk surface biological and physical parameters. The Spearman's rank correlations between GTE and in situ water temperature, chlorophyll a concentration, and CDOM absorption at 443 nm retrieved from the MODIS satellite were -0.25 (N=1061), -0.22 (N=991), and -0.21 (N=622), respectively. The CDOM data were downloaded from https://oceancolor.gsfc.nasa.gov/l3/, which is maintained by NASA Goddard Space Flight Center, Ocean Ecology Laboratory, Ocean Biology Processing Group (ref. [7]).

The absence of a clear relationship between GTE and phytoplankton is illustrated by the cruise track near the South Sandwich Islands (Supplementary Figure S7). During the outward leg of the transect, the ship encountered and mapped a phytoplankton bloom with high

chlorophyll a concentration (up to 1.6 µg L^{-1}), low fCO_2 (down to 300 µatm), and low GTE (down to 0.52). Spatial patterns in GTE and in chlorophyll a did not show a simple relationship of 'higher chlorophyll = lower GTE'. On the return leg of the transect to approximately the same location three weeks later, the phytoplankton bloom had concluded and GTE was higher at around 0.58. These observations suggest that surfactants may be made up of biologically labile organic compounds with relatively short lifetimes, and their activity in the surface ocean cannot simply be related to phytoplankton biomass.

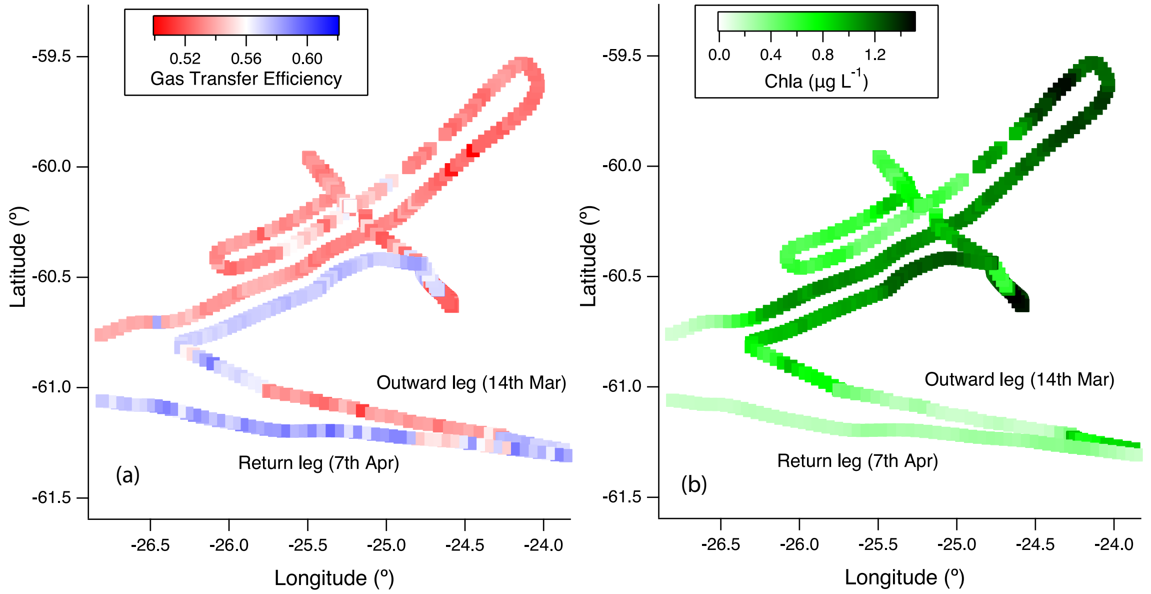

Figure S7. Map of cruise track near the South Sandwich Islands, color-coded by a) the gas transfer efficiency (GTE), and b) chlorophyll a concentration. This example illustrates the variability in the GTE and its lack of obvious relationship with chlorophyll a.

References

1. Edson, J. B. et al. On the exchange of momentum over the open ocean. *J. Phys. Oceanogr.* **43,** 1589–1610 (2013).

2. Butterworth, B. J. & Else, B. G. T. Dried, closed-path eddy covariance method for measuring carbon dioxide flux over sea ice. *Atmos. Meas. Tech.* **11,** 6075–6090 (2018).

3. Wohl, C. et al. Underway seawater and atmospheric measurements of volatile organic compounds in the Southern Ocean. *Biogeosciences*. **17,** 2593–2619 (2020).

4. Butterworth, B. J. & Miller, S. D. Air–sea exchange of carbon dioxide in the Southern Ocean and Antarctic marginal ice zone. *Geophys. Res. Lett*. **43,** 7223–7230 (2016).

5. Edson, J. B. et al. Direct covariance measurement of CO2 gas transfer velocity during the 2008 Southern Ocean Gas Exchange Experiment: Wind speed dependency. *J. Geophys. Res.* **116**, C00F10 (2011).

6. Landwehr, S. et al. Using eddy covariance to measure the dependence of air-sea CO2 exchange rate on friction velocity. *Atm. Chem. Phys*. **18,** 4297–4315 (2018).

7. NASA Goddard Space Flight Center, Ocean Biology Processing Group: MODIS-Aqua Absorption due to gelbstoff and detrital material at 443 nm, GIOP model, Accessed 2021/05/19. Maintained by NASA Ocean Biology Distributed Active Archive Center (OB.DAAC), https://oceancolor.gsfc.nasa.gov/data/aqua/ (2021).
